# A nonrandomized interventional study on perioperative fluid in children

**DOI:** 10.4103/0971-9261.70652

**Published:** 2010

**Authors:** Cenita J. Sam, Pavai Arunachalam, M. Sivamani

**Affiliations:** Department of Pediatric Surgery, PSG Institute of Medical Science and Research, Coimbatore, Tamilnadu, India; 1 Department of Community Medicine, PSG Institute of Medical Science and Research, Coimbatore, Tamilnadu, India

Sir,

There have been reports of postoperative deaths due to hyponatremia in children after surgery, such as tonsillectomy, orchiopexy and appendectomy. All these children had received hypotonic fluids such as Isolyte P.[1,2] Following these reports, the National Patient Safety Alert was issued in 2007 by NHS.[3] Because of the secretion of ADH in the perioperative period, isotonic fluids should be administered and may have to be reduced to two-third the requirement when they are very sick.[2–4]

Administration of dextrose was thought to be mandatory as it was difficult to detect hypoglycemia in an anesthetized child.[4] But, hyperglycemia causes osmotic diuresis, dehydration and electrolye imbalance. The danger of hyperglycemia in the perioperative period is a real clinical issue that has been extensively reviewed.[5]

We have conducted a prospective, nonrandomized study after obtaining approval from the ethical committee. The aim of the study was to estimate the postoperative sugar levels after administering 5% sugar/sugar-free fluid and to note the incidence of hypoglycemia and hyperglycemia. Children undergoing surgery were enrolled and were divided into two groups. Group I received sugar-containing fluids (5% sugar) and Group II received sugar-free fluids (RL or NS). Fasting was as per the ASA guidelines and fluid was started at the time of surgery. In Group I, the blood sugar level was estimated 4 h after surgery. In Group II, the blood sugar level was estimated immediately after surgery and again after 4 h. Serum sodium was estimated after 4 h in Group II.

Twenty-nine patients were enrolled in Group I and 39 patients in Group II. The mean sugar level was 121.03 ± 34.11 mg/dl in Group I and 107.33 ± 17.42 mg/dl in Group II at 4 h, *P* value = 0.054. Eight patients (27.5%) developed hyperglycemia, with a mean value of 166.88 ± 32.53 mg/dl in Group I and six patients (15.38%) had hyperglycemia, with a mean value of 135.16 ± 5.07 mg/dl in Group II. The *P*-value was 0.028. It was seen that there was hyperglycemia (48.5% immediately after surgery and 15.38% after 4 h) even after the administration of sugar-free fluid. The level of hyperglycemia was higher after the administration of 5% sugar-containing fluid.

Group II had their sugar tested immediately after surgery as they had received sugar-free fluid, and there was no hypoglycemia. The lowest sugar level was 80 mg/dl immediately after surgery and 72 mg/dl after 4 h. Hence, administering sugar-free fluid did not cause hypoglycemia in the immediate postoperative period.

Serum sodium was estimated in Group II as RL and NS contain more than the recommended amount of sodium. The mean was 137.94 ± 1.49 mEq/L. In spite of administering an increased amount of sodium, there was no incidence of hypernatremia.

We conclude that the administration of isotonic fluids is safe in the perioperative period as it causes only mild hyperglycemia and no hypoglycemia. This is also not associated with hypernatremia. Administration of 5% sugar-containing fluids is associated with hyperglycemia.
